# Community engagement and financial arrangements: Navigating institutional change

**DOI:** 10.1017/cts.2023.683

**Published:** 2023-11-16

**Authors:** Linda Sprague Martinez, Riana C. Howard, Marieka Schotland, Rebecca Lobb, Tracy Battaglia, Susan Stone, Coco Auerswald, Emily Ozer

**Affiliations:** 1 Boston University School of Social Work, Boston, MA, USA; 2 Boston University Clinical Translational Science Institute, Community Engagement Program, Boston, MA, USA; 3 University of California Berkeley, School of Public Health, Berkely, CA, USA; 4 Boston University School of Medicine, Boston, MA, USA; 5 Boston Medical Center, Boston, MA, USA; 6 University of California Berkeley, School of Social Welfare, Berkely, CA, USA

**Keywords:** Community engagement, financial arrangements, subcontracting, institutional barriers to translational science

## Abstract

Despite their documented benefits, the widespread adoption of community-engaged and participatory approaches among health researchers remains limited. Institutional practices and policies influence the uptake of community engagement and participatory approaches. We examine the role of financial arrangements between university researchers and community partners, by exploring efforts to bridge the gap between research administration and researchers at two research-intensive institutions. The type of financial arrangement a researcher has with a community partner plays an important role in setting the stage for the structure of the partnership as it relates to shared decision-making and ownership of the research. Continued efforts to clarify and streamline subcontracting processes are needed as is infrastructure to support community partners and researchers as they navigate financial arrangements if progress is to be made.

Community engagement (CE) and participatory research approaches have been identified as effective strategies to advance research translation [1,2]. Engaging diverse community stakeholders in the co-construction of research and in the development of the research process can facilitate the translation of evidence into practice and policy, facilitating uptake and adoption [3]. Those who integrate community-engaged and participatory approaches have identified several barriers, including resource distribution and financial arrangements [4–6]. This paper makes two important contributions to the literature. First, we outline key information on different financial arrangements as well as implications of these arrangements for power-sharing. Second, we demonstrate that incremental change in universities is possible with leadership by community engagement centers or individual investigators.

A nascent literature on structural governance in community-based participatory research speaks to the importance of shared decision-making as well as resource sharing [7,8]. We provide a framework for understanding the (a) different types of financial arrangements typically used to share funds across academic and community partners and (b) potential implications of these arrangements on community control and decision-making in research. This is an important starting point for advancing structural governance. As long-time academic investigators, we have observed that we, our colleagues, and our community partners typically do not understand the parameters and implications of different payment arrangements. Partnerships suffer when we lack capacity to effectively navigate administrative processes. Even the most well-intended researcher can end up with a fiscal relationship with limits on community ownership and decision-making, which threatens the kind of deep partnership required for participatory research approaches. Further, the time-sensitive nature of applying for funding can influence how we navigate determining the type of fiscal relationship to enter. Doing what is quickest in the grant preparation phase can lead to fiscal relationships that cause economic hardships for partnerships, and undermine partnership trust and collective impact [9].

Through two case examples, we illustrate institutional efforts at two distinctive research-intensive (“R1”) universities to align financial systems with community-partnered research. We first describe the work of the Clinical Translational Science Institute (CTSI) Community Engagement program, at Boston University (BU), a private institution, which is designed to build community engagement capacity. We then describe an extramurally funded initiative at the University of California Berkeley, a large public institution, designed to support a Research-Practice Partnership while strengthening the institutional conditions for community-partnered scholarship. Themes from the cases are discussed in the context of the literature.

## Types of Financial Arrangements

**Figure 1.**
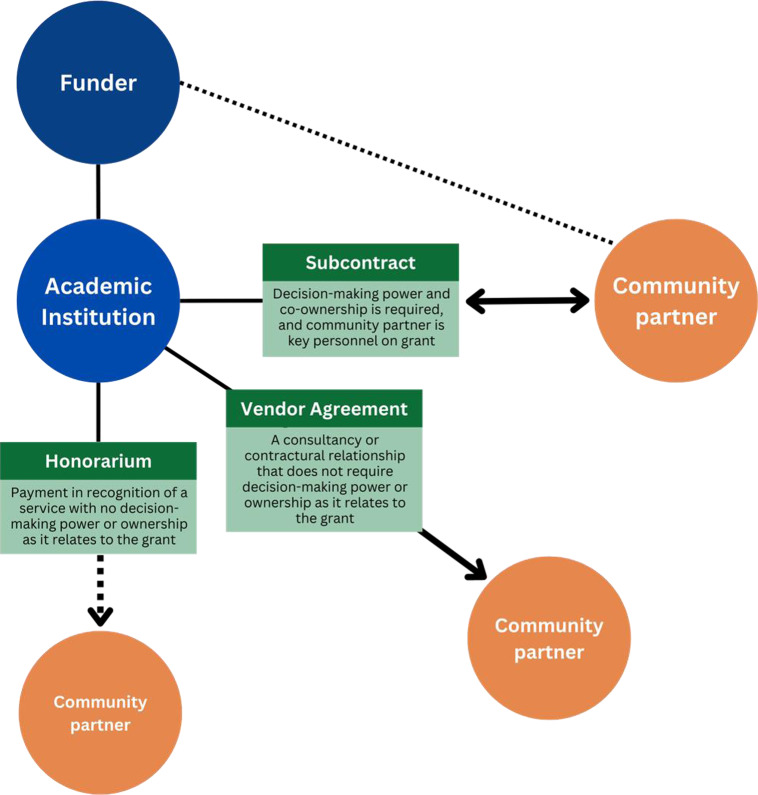
Types of financial arrangements and academic relationships.

There are multiple forms of financial arrangements that researchers engage in with community partners. Each type of arrangement signals the expectations of the partner in project decision-making and ownership [10]. Thus, the partners’ role in the study should inform the type of financial arrangement entered. Financial relationships have implications for the time researchers and community partners must allocate for administrative processes [11]. There are defined administrative guidelines for each form of financial arrangement. Increased ownership and scope necessities a greater level of financial oversight and thus, more paperwork and monitoring. Researchers who seek less burdensome mechanisms may unintentionally limit community partner ownership over the research. Honoraria, independent contracts, and sub-contractual arrangements (“sub-awards”) are common ways to compensate community members with a role in research beyond that of a research participant. Each is discussed and illustrated in Figure 1.

### Subcontract

A subcontract is a financial arrangement in which the community partner becomes a subrecipient of the award. Subcontracts are made to community investigators who have programmatic responsibility and decision-making authority over the direction of the project. When a community partner is a subrecipient, it means that they have been designated as key personnel on the grant, and have co-ownership over the project as outlined in their scope of work and in their budget justification. As such they are also expected to coauthor publications on project findings [12,13]. This form of financial arrangement is well aligned with deeply collaborative forms of research such as community-based participatory research, with respect to shared decision-making and ownership. However, the subcontracting process can present challenges for the research and community partner, particularly with small grassroots organizations. In addition to submitting paperwork including a letter of intent, scope of work, budget and justification, and biosketches, subrecipients are asked for a DUNS number (a numeric identifier obtained through Dun and Bradshaw) which requires completing a registration process [14]. In the case of federal grants, they must also register with sam.gov. This process can be time-consuming for partner organizations; moreover, when researchers are not familiar with the process, it can cause delays. In addition, institutional procedures are time-intensive, embedded in bureaucratic processes, and not always clear to researchers or communicated to partners. Upon funding, subrecipients are asked to complete questionnaires to determine the level of financial risk the subcontract presents to the university. Limited federal grant experience, may require additional procedural action such as risk mitigation and monitoring plans [13]. Bureaucratic processes associated with subcontracting can preclude meaningful collaboration with community organizations, however, understanding required processes that universities researchers and community partners are required to navigate in one another’s settings can reduce tensions. When processes are unexpected and rushed they can feel oppressive and top-down as well as interfere with sense of agency. Early discussions about processes can clarify expectations and inform the establishment of feasible timelines.

### Vendor Agreements

The independent contract or consultancy involves a procurement or “vendor” relationship between the institution and an entity or an individual providing a specific service [14]. These arrangements can take varied forms depending on the amount of the award and its duration. Of note, because independent contracts operate in a competitive environment [15], it is implied that there are *other* entities beyond the community partner who could provide the service. Although for many this form of arrangement may, in some cases, be less cumbersome than a subcontract, the “fee for service” nature of this arrangement runs counter to the values of equity and power-sharing inherent in many academic-community research partnerships. Co-ownership is not implied in this arrangement; on the contrary, at least on paper, a “power over” relationship is implied with the contractor providing a service to the researcher. There is no fiscal relationship between the funding agency and the partner; thus, the community partner is removed from having a meaningful role in the grant, as consultancies are “not subject to the federal program [15].” In practice, community partners who are paid as consultants can develop memoranda of understanding with researchers that involve equitable power-sharing; however, co-ownership is not implied from a fiscal standpoint. In some cases, consultancies are easier to set up than subcontracts, placing less burden on partners, but they have less recourse if there is a dispute with the researcher. The level of burden is variable across institutions, consultancies can require having insurance as well as navigating electronic invoicing systems and payment processes; compensation difficulties have been associated with consultant contracts [16].

### Honorarium

An honorarium can be paid for a service for which there is not a fixed price [17]. In academic settings, such services might include speaking engagements, workshop participation, reviews, or advisory roles. Importantly, an honorarium is *not* payment for services rendered but rather a form of recognition of appreciation for service provided [17]. Thus, community partners can be paid honoraria in recognition of services such as advising. An honorarium does not imply shared decision-making or co-ownership and as is the case of a consultancy or independent contract, it would not be an effective way to equitably distribute resources. An honorarium does not involve a contractual agreement. Administering an honorarium involves the submission of paperwork to justify the payment as well as paperwork for IRS reporting. Some institutions require resumes and/or biosketches for speakers. The nature of documentation required can pose an added hurdle for researchers, such as when community partner credentials are scrutinized and do not meet criteria as demonstrating expertise according to typical “professional” standards. This can impede the partnership process and cause tensions between financial and accounting professionals and researchers. For example, in the case of youth-led research and evaluation, high school students may be engaged as partners based on their lived experience of a health issue or system, as well as for their social networks and potential as changemakers. However, academic institutions and administrators may not view youth as experts or understand the need to compensate youth for their expert advice.

In sum, the type of financial arrangement a researcher has with a community partner plays an important role in establishing structural governance. Each form of contractual arrangement has a unique set of administrative processes and rules for the researcher and community partners to navigate. Although in some cases subcontracts are avoided to limit front-end complications, there may be unintended consequences associated with said avoidance.

## Strategies to Mitigate Financial Barriers to Engagement: Two Illustrative Cases

We describe two cases from our own practice designed to address financial and administrative barriers to CE and participatory research approaches. We outline the institutional context as well as the impetus for change and then detail the strategies employed and outcomes to date.

### Case 1: BU CTSI Community Engagement Program

The National Institutes of Health launched the Clinical and Translational Science Award (CTSA) initiative in 2006 to facilitate research translation through infrastructure development [18]. Today the CTSA program continues to “support a national network of medical research institutions …that work together to improve the translational research process to get more treatments to more patients more quickly [19].” Because people in communities know what they need to be healthy, each CTSA hub has a CE program designed bridge academic institutions and the broader community to help researchers to “understand what residents need” to be healthy [20].

The BU CTSA CE program has three aims focused on capacity building, partnership development, and dissemination [21]. Capacity-building activities are focused on both increasing researcher and community partner preparedness for community engagement and participation as well as increasing institutional capacity to support research teams with partnership development. Partnership activities, meanwhile, involve the establishment of structures to facilitate partnerships. In 2021, the CE Program launched a Community Engaged Research Speaker Series designed to increase dialog around CE best practices and to address institutional barriers to CE. In this case, we describe conversations designed to address financial barriers.

#### Strategy

We convened administrative leaders of financial departments to encourage open dialog about challenges experienced by community and researchers with the financial systems and to identify opportunities to minimize the challenges. We used a vignette to illustrate the examples of financial barriers. An underlying premise of this work is a recognition of the complexity of institutional change. This is particularly true in the case of universities which are complex systems, made up of multiple actors (researchers, students, administrators, staff) who operate in webbed networks both within and across silos [22]. As such, we set out to identify actors and build new relationships with the goal of increasing transparency and information sharing. We did this by identifying financial administrators in sponsored programs and accounting, understanding how they operate as a unit and how their unit fits into the larger system and interacts with school financial administrators and grants management [22].

We invited leaders from sponsored accounting on the main campus, the medical school campus, and the hospital affiliated with our university to participate in a panel discussion on financial procedures for research. We co-developed learning objectives for the panel discussion. During an initial planning meeting, the program goals were discussed. Learning objectives were developed that included: (1) understanding the administrative structures at the university involved in subcontracting with community partners and processing payments and (2) being more aware of the systems department finance personnel must navigate as they work with researchers to process subcontracts and payments.

A vignette was then prepared by the CE program illustrating examples of financial barriers encountered by researchers and community partners. The vignette and associated questions were shared with finance leaders on the panel in advance of the event. The session panel was advertised to researchers, community partners, and administrative staff from finance and sponsored accounting. A facilitator from the CE program posed vignette-based scenarios, which the panelists collectively were asked to troubleshoot. Panelists discussed how to determine the type of financial arrangement during the grant writing process and walked participants through the process of setting up an award for the community partner. The discussion included granular details such as the timing between submitted invoices and payments to the community partner and the system behind this process on the finance side. During the discussion, panelists explained the role of actors in their respective units and how they interface with the award and other relevant departments in the institutions. Through the discussion, the audience gained a deeper understanding of the procedures as well as the purpose of said procedures. Panelists also shared successful strategies they have employed to facilitate the establishment of financial arrangements as well as community partner payments. These strategies included (1) having a clear understanding of the roles and responsibilities of the partners and (2) beginning conversations with finance early in the grant writing process to allow time for planning and the completion of required paperwork.

#### Outcomes

A total of 19 participants from across five schools, the government and community affairs office, and the medical center participated in the event. Participants included faculty, researchers, research staff, and administrative and finance staff. During the event, we heard from researchers that it was helpful to learn more about finance processes and the reasoning behind them. In discussions with panelists following the event, we were directed to additional resources for researchers and trainees including how to ascertain additional information about the finance processes. In addition, panelists were interested in continuing conversations with our team and thinking about ways to get information about financial processes to researchers.

The community engagement program incorporates resources related to financial processes in consultations and training. Our next steps are to deepen our relationship with sponsored accounting and to continue to promote their resources with academic and community stakeholders. In addition, as financial barriers are identified our programs will be sharing them with sponsored accounting to identify strategies to make our systems more accessible. During the session, budget development also emerged as a topic. Given many researchers and trainees do not engage in participatory budgeting with partners we are also integrating more information about this process into our programing.

#### Lessons learned

As researchers, we are accustomed to working with one financial contact in our respective schools. During this process financial administrators learned more about the fiscal challenges experienced by CE researchers and researchers learned the person whom they typically work with is part of a web of financial administrators and staff whose work is shaped by both institutional and government rules and regulations.

### Case 2: Institutional Change at UC-Berkeley

UC-Berkeley, the oldest university in the 10-campus University of California public system, has a long tradition of research in the public interest and in the sustaining of research partnerships with local, state, national, and international governmental and non-governmental entities. Berkeley is a large campus, with more than 2700 faculty and approximately 8000 staff and administrators. In 2015, a network of over 20 faculty investigators, across disciplines and academic units, all with research focused on promoting adolescent wellbeing and equity through collaborative research, organized into the Innovation for Youth (i4Y) Center. Within i4Y, we noted that many of us shared struggles in navigating the university bureaucracy as we conducted community-partnered research. Despite Berkeley’s strong public mission, we encountered myriad “pain points'' that can undermine academic-community partnerships and our capacity to conduct research aligned with our public mission.

#### Strategy

We, in partnership with our SF Unified School District partners, successfully applied for a WT Grant Foundation Institutional Challenge Grant (co-funded by Doris Duke Charitable Foundation) that supported our Research-Practice Partnership *and* our efforts to push UC-Berkeley to make institutional changes to better support community-partnered scholarship. While our focus here is on the domain of financial arrangements, we are also actively working on institutional policies and practices to (a) strengthen recognition of community-partnered research in faculty evaluation and (b) collaborate to address pain points in the domains of the IRB; Sponsored Projects Office; and intellectual property and data-sharing. The extramural grant was a key catalyst for our campus change work, especially in the domain of faculty evaluation, where, with critical support of the faculty senate leadership brokered by the Associate Vice Chancellor for Research, we achieved new campus-wide policy guidelines to recognize and credit community-engaged scholarship [23].

The path to engaging with pain points related to financial arrangements was less clear, however, perhaps because administrative financial systems are outside of faculty governance, often experienced by investigators as mystifying, and engage multiple administrative units with distinctive reporting lines across campus. For example, when we as faculty investigators feel frustrated by the inability to pay community partners in a timely way, we typically don't understand exactly why or where a payment is “stuck.” Is it a routine administrative delay in an overstressed financial system? Without adequate knowledge of different financial arrangements, did we unwittingly go through “a wrong door” by trying to use an ill-fitting financial arrangement? Are our campus systems set up to be so risk-averse that to avoid liability there is *too much* oversight and cross-checks on low-risk research activities? The stakes are high, as the consequences for payment delays can be major, especially for low-income partners. Delays on honoraria payments for senior faculty are unlikely to cause hardship, but delayed honoraria for unstably housed or food-insecure young people do. In our pain points memos, several faculty members shared that they sometimes felt driven to pay out of pocket without reimbursement to keep promises to under-resourced youth partners and without staff support to navigate reimbursement systems.

In addition to extramural funding, our primary strategy was investigator and administrative stakeholder engagement. First, drawing on our existing networks within i4Y and across campus, we invited investigators to identify pain points for partnered research in a shared online document. We further engaged faculty and staff in 1:1 meetings and larger convenings to collect information; we also shared “pain points” memos with our community co-investigators for input. We circulated memos as widely as possible, including with our Vice Chancellor for Research Office and other campus leaders (and added to our I4Y website in 2022). We thus engaged in a range of “bottom-up” efforts to advocate respectfully for innovations, acknowledging the systemic nature of the problems, faculty’s own need for education, and that administrative staff has been stretched thin by budget cuts and the pandemic. In this organizing effort, our change team was positioned outside of the administrative systems, without direct power, but with network access due to co-PI Stone’s position as a tenured senior faculty member and Associate Dean of Social Welfare, and co-PI Ozer’s position as a tenured senior faculty member (and in 2022 as Faculty Liaison to the Provost on Public Scholarship and Community Engagement).

#### Outcomes

After two years of information-gathering and framing of the problem, in 2022 we were able to form a coalition with a nascent effort with similar goals led by the deans of the public policy school. We joined forces with this group who, working with senior staff experts, called on campus leaders to support a pilot effort to streamline frustrating pain points for partnered scholarship. Around the same time, the incoming Provost highlighted combating bureaucracy–generally, not specifically related to community-partnered scholarship–as a major goal, initiating a broad task force with faculty and campus leadership. As of Spring 2023, our cross-unit pilot coalition is providing momentum for several concrete changes to create more flexibility in financial arrangement rules, via organized working groups with the support of the Vice Chancellor for Research Office and the Assistant Vice Chancellor for Business Administration. It is critical that these working groups include long-time staff who are familiar with the specific financial and administrative processes, as well as tenured faculty who enjoy job security outside the administrative hierarchy and thus feel highly empowered to raise concerns. We have yet to achieve our desired policy outcomes, but we have seen important shifts in awareness, communication patterns, and social networks across the levels of our large campus, as well as significant time commitments on the part of staff and faculty to engage in the change process (no small achievement given competing demands).

#### Lessons learned

While we are early in our process, lessons learned include (a) the importance of identifying and sharing templates for smoother financial processes and to address valid risk management concerns, (b) engaging collaboratively and respectfully with administrative colleagues as allies in demystifying processes, (c) identifying a feasible scope of effort given the complexity of systems, and (d) addressing that long-time staff, investigators, and community partners may feel pessimistic about the possibility of change, having experienced prior efforts that failed. As we move forward with this work, we aim to identify which aspects of financial arrangements that cause friction for community-partnered scholarship are “fixed” versus more flexible, i.e. what are the “load-bearing” essential elements required for legal and compliance issues, versus those that may represent interpretations on the part of our administrative systems, or unnecessary replication of compliance checking for low-risk activities. We aspire to have clearer templates and guidelines to have smoother paths for community-partnered research, and strengthen the support for community partners and investigators as they navigate our systems (i.e. a potential concierge model), while broader campus efforts seek to promote an administrative culture that has “no wrong doors.” Beyond this current push, a key challenge is sustainability: Where will these efforts live and how will they be integrated into the governance and accountability routines of the institution moving forward?

## Discussion

Consistent with the literature, researchers and community partners at both institutions described frustration with what often seemed like impossible processes to navigate in a timely way [11]. We note that timeliness is a key aspect of community-partnered scholarship, as the research questions pursued need to be responsive to real-time policy and practice decisions. We’ve learned that (1) **i**nvestigators are often unaware of payment processes and may seek to identify the easiest way to pay partners without a deep understanding of the contractual implications for shared decision-making; and (2) finance departments know little about community-engaged and participatory research but are receptive to engaging in dialog with researchers. Continued efforts to clarify and streamline subcontracting processes are needed, given most research funding is awarded directly to academic institutions, not community-based organizations [23].

Research institutions–once funded–are charged with the redistribution of financial resources which can further strain partnerships. Consistent with the literature, we have found that layered administrative processes can produce cumbersome payment systems that are difficult to navigate for both researchers and community partners. Thus, the compensation process can feel convoluted and be time-intensive [11]. Moreover, prolonged delays can result in frustration, which can threaten relationships between partners, particularly in cases in which trust has not yet been established. Delayed payments can also stymie the research process altogether.

We see that complications associated with community partner compensation are driven by multiple factors. Universities are large bureaucratic institutions with multiple layers each with its own unique processes [22]. Institutional departments that govern financial processes and expenditures are often removed from the purview of the researcher who may or may not have a deep understanding of said processes. The ability to navigate institutional processes is essential for moving financial arrangements and compensation forward. University systems are siloed. As such, researchers and financial and accounting departments can have limited opportunities for dialog and relationship building. The lack of network ties between these actors can limit information sharing between these groups, yet their work is interdependent [24]. Moreover, research training often does not include content related to financial arrangements and administrative processes and administrators are not trained in CE or participatory research. Although research administration provides guidance for researchers, engagement with and awareness of these resources can vary. The divide between research administration and researchers can inhibit the governance of CE, and as such is a threat to translational science.

## Conclusions

Alleviating administrative burden is complex and multifaceted. Small wins are important for sustaining momentum while navigating systems change. Our work related to financial systems has been primarily about trying to make things work more smoothly. Although it can feel like systems are broken, they are in fact operating as they were intended to operate, without community participation in mind. As such, developing a deep understanding of financial and administrative processes among researchers is a critical first step in finding ways to create structures that facilitate community partnerships. However, it is not enough. There is a need for stronger infrastructure to support community partners and researchers as they navigate financial arrangements if progress is to be made.
